# Differential Hypothalamic-pituitary-adrenal Response to Stress among Rat Strains: Methodological Considerations and Relevance for Neuropsychiatric Research

**DOI:** 10.2174/1570159X21666221129102852

**Published:** 2023-07-10

**Authors:** Antonio Armario, Xavier Belda, Humberto Gagliano, Silvia Fuentes, Patricia Molina, Sara Serrano, Roser Nadal

**Affiliations:** 1 Institut de Neurociències, Universitat Autònoma de Barcelona, Barcelona, Spain;; 2 Traslational Neuroscience Unit, UAB-Parc Taulí, Sabadell, Spain;; 3 Department of Cellular Biology, Physiology and Immunology, Animal Physiology Unit, Faculty of Biosciences, Universitat Autònoma de Barcelona, Spain;; 4 CIBERSAM, ISCIII, Madrid, Spain;; 5 Department of Psychobiology, Faculty of Psychology, Universidad de Granada, Granada, Spain;; 6 Psychobiology Unit, Faculty of Psychology, Universitat Autònoma de Barcelona, Barcelona, Spain

**Keywords:** Hypothalamic-pituitary-adrenal axis, strain differences, anxiety, depression, coping, ACTH, corticosterone, corticotropin-releasing hormone

## Abstract

The hormones of the hypothalamic-pituitary-adrenal (HPA) axis, particularly glucocorticoids (GCs), play a critical role in the behavioral and physiological consequences of exposure to stress. For this reason, numerous studies have described differences in HPA function between different rodent strains/lines obtained by genetic selection of certain characteristics not directly related to the HPA axis. These studies have demonstrated a complex and poorly understood relationship between HPA function and certain relevant behavioral characteristics. The present review first remarks important methodological considerations regarding the evaluation and interpretation of resting and stress levels of HPA hormones. Then, it presents works in which differences in HPA function between Lewis and Fischer rats were explored as a model for how to approach other strain comparisons. After that, differences in the HPA axis between classical strain pairs (*e.g*. High and Low anxiety rats, Roman high- and low-avoidance, Wistar Kyoto *versus* Spontaneously Hypertensive or other strains, Flinder Sensitive and Flinder Resistant lines) are described. Finally, after discussing the relationship between HPA differences and relevant behavioral traits (anxiety-like and depression-like behavior and coping style), an example for main methodological and interpretative concerns and how to test strain differences is offered.

## 1 INTRODUCTION

### 1.1 The Hypothalamic-pituitary-adrenal Axis and the Stress Response

Although there is no full agreement regarding the definition of stress, we consider one of the most appropriate that proposed by Vigas [1]. He defined stress as the response of the organism, which evolved in the course of phylogeny, to agents actually or symbolically endangering its integrity. This definition includes both systemic and emotional stressors. Systemic stressors refer to situations implying real harm or the need for strong metabolic demands to the organisms, which cannot be solved only with normal homeostatic mechanisms. Emotional stressors include situations that are not harmful on their own but predict a certain possibility of real danger or strong metabolic demand. These two types of stressors lead to a reactive or anticipatory response, respectively, and are differentially processed by the brain [2]. We prefer the terms systemic-emotional instead of physical-psychological to avoid mind-body dualism. Exposure to stressors triggers a myriad of biological changes in the organism, but we will mainly focus on the hypothalamic-pituitary-adrenal (HPA) axis.

Glucocorticoids (GCs) are the final hormones of the HPA axis and the most extensively studied stress hormones. The prominent role of GCs in stress research is due to their widespread and critical function in an important set of physiological and behavioral consequences of exposure to acute and chronic stress. However, the response to stress is extremely complex and the activation of the HPA axis with the final release of GCs (cortisol in humans and most mammals, corticosterone in rats and mice) is only one aspect of such response. Consequently, there are other important biological players. Although the present review will focus on the HPA axis, other physiological responses, particularly hormones, will be commented on when appropriate.

Despite the agreement regarding the adaptive role of GCs to cope with stress, the wide range of actions and organs affected by GCs has made it difficult to delineate a clear picture of their role in stress. Nevertheless, considering the most common stressful situation in nature, coping with the presence of a predator, it is unlikely that GCs play a critical role to solve the present situation. The presence of a predator can elicit different behavioral responses depending on the distance to the predator (from long to short): flight, freezing or fight. The flight-fight response might require immediate and intense physical activity, but GC release is only evident 5 min after stress starts, with the maximum at 20-30 min. Therefore, the immediate response to the presence of a predator is critically dependent on sympathetic activation and the release of catecholamines rather than on GCs. GCs might, in turn, be relevant to cope with prolonged (hours) exposure to stressors (gluconeogenesis), to recover from the present situation, and to prepare for future situations [3], including the memory of the previously encountered stressor [4].

It is well-known that there are important individual differences in the physiological and behavioral consequences of exposure to stress in all species. However, the precise mechanisms involved in such differences are poorly known. The characterization of individual differences in the neuroendocrine response to stress is an important issue for several reasons. First, some classical stress hormones, particularly those of the HPA axis (ACTH and cortisol/corticosterone) and prolactin, are among the few biological markers that have been demonstrated to be sensitive to the intensity of emotional stressors [5]. Therefore, a greater response might suggest an enhanced vulnerability to stress. Second, stress-induced GC release is one of the major mediators of the physiological and behavioral consequences of stress. Thus, an inappropriate response (lower or higher than normal) is likely to contribute to the negative consequences of stress [6-8]. Third, altered neuroendocrine responsiveness might be the reflection of alterations in neurotransmitters and circuits regulating such responsiveness, and these abnormalities might extend to other circuits also involving the same neurotransmitters. Not surprisingly, there has been over decades a marked interest in the possible alterations of the HPA axis in psychiatric diseases, including unipolar and bipolar depression, schizophrenia, anxiety disorders, and posttraumatic stress disorder, although the picture is far from clear [9].

To better understand the putative meaning of such differences, a brief outlining of the HPA axis is needed [10, 11]. The key area in the control of the HPA axis is the paraventricular nucleus of the hypothalamus (PVN), particularly the medial parvocellular dorsal subdivision (mpdPVN) where neurons synthesizing the corticotropin-releasing factor or hormone (CRF or CRF) are mainly located. These neurons project to the pituitary portal blood of the median eminence, where CRH is released to reach the anterior pituitary corticotrope cells. CRH acts in corticotrope cells through CRH type 1 receptors (CRHR1) to induce the synthesis of the ACTH precursor proopiomelanocortin (POMC) and the release of ACTH into the bloodstream. Vasopressin acts on corticotrope cells favoring the effects of CRH on ACTH release. This is of interest because some mpdPVN CRH neurons also co-express vasopressin and the number of double-positive neurons increased after chronic hypersecretion of ACTH caused by either adrenalectomy or chronic stress exposure [12].

ACTH acts through melanocortin type 2 receptors in the cells of the zone fasciculata of the adrenal cortex to induce the synthesis and release of GCs. It is important to note that some factors other than ACTH are capable of activating the adrenal without parallel changes in circulating ACTH release or can modulate adrenal sensitivity to circulating ACTH. The best characterized of such factors is the sympathetic innervation of the adrenal gland, which is presumably involved in the circadian changes in adrenal sensitivity to ACTH and the dissociation between ACTH and corticosterone observed after exposure to prolonged stress [13].

The activity of the HPA axis is subjected to negative feedback by corticosteroids that constrains both resting and stress levels of ACTH [14]. This negative feedback is exerted through the concerted action of mineralocorticoid (type I; MR) and glucocorticoid (type II, GR) receptors acting at the corticotropes and different brain levels, including the PVN, the hippocampal formation, and the medial prefrontal cortex [14]. Negative feedback involves both non-genomic fast effects and more delayed genomic mechanisms. Although suppression by the synthetic glucocorticoid dexamethasone (DEX) is typically used in most studies in humans and animals, its use has been questioned as its access to the brain is limited by the multidrug-resistant protein 1 (MDR1) that excludes DEX from the brain to a higher extent than natural GCs [15, 16]. Corticosterone is even less excluded than cortisol [15, 16]. As a consequence, the effect of DEX, in contrast to natural GCs, is mainly exerted at the level of the anterior pituitary corticotrope cells.

### 1.2 Studying Strain Differences in the HPA Axis: Resting and Stress Levels: General Overview

Interest in the characterization of individual or strain differences in the activity of the HPA axis has focused on resting levels or, more frequently, on its responsiveness to stressors. Both topics are of potential interest. A nice description of the main aspects to be considered when assessing the activity of the HPA axis can be found in Spencer & Deak’s review [11]. Circulating levels of GCs show a marked rhythmicity characterized by both pulsatile secretion and circadian fluctuations. The latter is strongly associated with the daily pattern of activity. Thus, in humans, the highest levels are observed after awakening or in the next hour and the lowest levels in the first sleeping hours, whereas in rats (and mice), low levels are observed at lights on and the highest levels around lights off. The pulsatile and circadian nature of glucocorticoid secretion has important methodological consequences when trying to characterize individual differences. Regarding circadian rhythms, blood levels need to be evaluated at different times of the day to know whether overall secretion is altered or alterations are restricted to specific daytimes. This is an important issue as it is now acknowledged that a flattened circadian glucocorticoid rhythm might have functional consequences, even if overall secretion is not altered. Pulsatile secretion implies that blood levels might markedly differ within a particular individual from moment to moment. Therefore, a unique sample is far to be representative of individual differences. These problems have been reduced in humans by using the aggregated data of samples taken on various days [17], and the same strategy has been adopted by our laboratory in rats recently [18]. Using this strategy, more representative values are obtained. Suppose strains rather than individual differences are evaluated. In that case, aggregated data are not needed, but still obtaining samples at various times across the day would be required to detect overall secretion and possible changes in the circadian rhythm amplitude.

Regardless of the number of samples obtained, a major concern when reviewing available literature is the apparent difficulty to obtain true resting levels of HPA hormones. In our hands, with radioimmunoassay (RIA) procedures, true resting levels of corticosterone in male rats are 10-20 ng/ml at the nadir and 150-200 ng/ml at the peak. An inspection of the literature reveals that most of the studies report values considerably higher, particularly at lights on. Corticosterone levels are extremely sensitive to minor perturbations in the animal room and most animal facilities are not designed to work in stress. It could be argued that all animals or strains are exposed to the same perturbations and therefore, results are representative of subject/strain differences. However, there are multiple-examples of normal resting activity but altered responsiveness to stress. Consequently, we can erroneously interpret strain differences in stress responsiveness as strain differences in resting HPA activity. Obviously, strains might often differ in both aspects.

The problems to obtain true resting levels of HPA hormones are due to a combination of some critical aspects. First, noise or any other perturbation in the animal room caused by construction near the animal facility [19], by unexperienced animal caretakers in the period preceding the experiment or by inexperienced or poorly trained researchers entering the animal room and touching the cages [20]. Second, the time elapsed between taking the cages and blood sampling: simply moving a cage to another place can alter hormone levels; also touching the cages and taking one animal can alter corticosterone levels in the animals remaining in the cages that would be sampled later [21]. This is particularly relevant in group-housed animals. Finally, the method of blood sampling: true basal levels are obtained by the tail-nick procedure as compared to rapid decapitation [22] if researchers have experience with the procedure. In contrast, all anesthetics except perhaps pentobarbital are known to strongly activate the HPA axis [10] and this should always be avoided.

What about possible concerns in the interpretation of the observed subjects/strain differences in HPA responsiveness? Again, the interpretation of the data is very often excessively simple or even partially erroneous for several reasons. It is important to consider the possibility that differences in stress responsiveness are due to vendors rather than to strains when not all animals are from the same vendor or breeding center [23]. Regarding variables measured, it is frequent that comparisons are restricted to blood levels of corticosterone, with the assumption that corticosterone would reflect ACTH release. However, it is well-known that adrenocortical secretion reaches a maximum with intermediate levels of ACTH and therefore is unable to reflect possible differences in ACTH when relatively strong stressors are used [5]. Only when stressors are of low intensity (*e.g*. open-field exposure) or blood corticosterone levels are followed after the termination of stressor exposure is corticosterone reflecting ACTH release. Moreover, individual or strain differences might exist in adrenocortical sensitivity to ACTH and therefore differences in corticosterone do not necessarily reflect differences in ACTH. Strain differences in other molecules participating in the activity of the HPA axis have been less explored. In this regard, blood levels of transcortin (corticosteroid binding-globulin) are particularly important. CBG levels determine the free levels of corticosterone, considered the biologically active fraction. As measuring free plasma GCs is technically demanding, measurement of total GCs together with CGB can give us an idea of free GCs fraction. An indirect way of detecting possible overall differences in corticosterone is thymus weight, which is very sensitive to circulating levels of corticosterone in rats [24]. Recently, hair corticosterone concentration has been pointed out as an integrated measure of free corticosterone levels over periods of a few weeks [25]. Hence, this parameter allows for detection, with a simple measure, of possible strain differences [26].

Another important consideration to characterize individual/strain differences is the type of stressor. If differences in stress responsiveness are the result of brain processing of stressors upstream of the PVN, the type of stressor is clearly relevant, particularly when comparing systemic *versus* emotional stressors. An example of the critical contribution of the type of stressors is the comparison of Roman strains reported by Gentsch and colleagues [27], who demonstrated higher ACTH response in Roman-low avoidance (RLA) than Roman-high avoidance (RHA) after exposure to relatively mild stressors but not in the case of severe stressors. In general, even within emotional or predominantly emotional stressors, the contribution of qualitative and quantitative aspects of stressors might be relevant.

If two strains markedly differ in basal and/or stress levels of ACTH, this is likely to be the consequence of differences in the inputs to the PVN or the responsiveness of parvocellular neurons synthesizing CRH and/or vasopressin. Expected canonical differences in those strains showing higher ACTH levels are a higher expression of CRH in the mpdPVN and of POMC in the anterior pituitary. If only subtle differences in ACTH levels are observed, it is possible that no differences in CRH and POMC expression are observed.

In some cases, the altered function of the HPA axis can be related to alterations in GCs negative feedback. This has been typically tested by injecting DEX and the interpretation of the results is problematic considering that DEX mainly acts at the level of corticotrope cells, not in the brain, as previously commented. Even if natural GCs are used, negative feedback can only be accurately assessed by measuring ACTH, not corticosterone, and the precise brain area involved in differential negative feedback cannot be delineated after systemic administration.

Possible differences in the HPA axis have been explored in various strains of rats and mice genetically selected for criteria not related to the HPA axis. In some cases, a pair of strains from a common origin were obtained that dramatically differ in a particular characteristic. It is not our intention to exhaustively review all these pairs of strains but to use some of them to illustrate how to investigate the HPA function. We will focus particularly on HPA hormones and the main hypothalamic secretagogue (CRH) and not on GCs receptors either in the periphery or the brain. A schematic illustration of the HPA axis and its regulation, as well as the main stimuli employed to test differences in this axis, is shown in Fig. (**1**). Importantly, we have tried to incorporate most results from the literature in the present review, but our purpose has been to be illustrative rather than exhaustive.

## 2 LEWIS-FISCHER RATS AS A REFERENCE FOR HOW TO APPROACH STRAIN DIFFERENCES IN THE HPA AXIS

Inbred Lewis rats are mainly characterized by a particular susceptibility to develop experimental arthritis after systemic injection of streptococcal cell wall (SCW) compared with the histocompatible inbred Fischer strain. It was soon realized that the two strains differed in HPA activity and this could be related to the differential susceptibility of Lewis rats to arthritis (see below). Consequently, the two strains have been extensively studied and constitute an example of possible alterations at different levels of the HPA axis. From the recent perspective of possible sex differences, the Lewis-Fischer models are of great interest as most earlier results were obtained in females, where the differential susceptibility to experimental arthritis is more prominent and was first described.

Although no differences in morning basal corticosterone levels between females of the two strains were originally reported [28], when the circadian pattern rather than a single time point was studied, lower corticosterone levels were observed in Lewis *versus* Fischer around the lights off-peak [ 28, 29].

Importantly, marked differences were found between females of the two strains in ACTH and corticosterone responses to different types of immune (SCW, interleukin-1α), systemic (ether) and emotional (open field, restraint, forced swim) stressors, with a lower response in Lewis rats [28, 30]. Similarly, a defective corticosterone response to another immune stressor (endotoxin) was further observed [31]. Impaired HPA response of female Lewis rats was not restricted to typical stressors and can also be observed in response to drugs known to activate the HPA axis through various neurotransmitter receptors, including muscarinic, adrenergic, and serotoninergic [28, 32]. The reduced response of Lewis rats to so many different stimuli strongly suggests that the main defect could be at the level of the PVN itself and probably could affect CRH gene expression and release. Supporting this, basal CRH gene expression did not differ between the two strains in the PVN, but a defective response to SCW, IL-1α and restraint was observed [30, 33] along with a reduced *c-fos* response to water avoidance stress [34]. Another study reported lower levels of basal CRH gene expression in the whole PVN associated with higher levels of vasopressin gene expression [35] and enhanced *in vitro* hypothalamic release of vasopressin [36]. The authors suggested that enhanced vasopressin expression and release could compensate for reduced CRH activity. However, these results illustrate an important problem regarding the possible role of vasopressin in the control of the HPA axis: the procedures did not distinguish between magnocellular vasopressin neurons that project to the neurohypophysis and parvocellular vasopressin neurons of the mpdPVN, which are those presumable involved in the control of ACTH release. Therefore, the functional meaning of these higher levels of vasopressin in Lewis rats remains unclear.

An alteration at the level of the PVN and CRH neurons does not preclude other downstream alterations. For instance, reduced *in vivo* ACTH response to exogenous CRH administration was also reported [28, 37], suggesting differences at the level of corticotrope cells in the anterior pituitary. To our knowledge, only one study has explored corticosterone responsiveness to exogenous ACTH administration to detect differences at the level of the adrenal cortex [38]. In that study, a lower lasting rather than lower maximum corticosterone response was observed in both male and female rats Lewis *versus* Fischer, tentatively suggesting impaired adrenocortical responsiveness in Lewis of both sexes.

Are the same strain differences observed in males? Whereas the above data in female rats are quite consistent, differences between males of the two strains are less obvious, although the overall data fit with results in females. Male Lewis and Fischer rats were first compared by Dhabhar and colleagues [39], who found lower basal corticosterone levels in Lewis than in Fischer at most times of the circadian rhythm, although levels were also higher in Fischer when compared with outbred Sprague-Dawley (SD) rats. Differences in the circadian rhythm between Lewis and Fischer were later confirmed [40, 41]. However, in those studies where only basal ACTH and/or corticosterone levels were assessed at a single time point in the morning, no differences were usually observed (*e.g*.) [42-44]. Despite no strain differences in morning basal levels, a lower HPA response to forced swim [43, 45], restraint [44, 46, 47], transfer in a bucket [48], noise [49], or morphine [50] was found in Lewis than Fischer. In contrast, no differences in basal or stress corticosterone levels were found between Lewis, Fischer, SD and Brown-Norway rats in response to foots-hocks [51], although the negative results could be due to adrenocortical saturation of corticosterone synthesis. This saturation can explain why, in response to restraint, lower corticosterone response of Lewis *versus* Fischer was only noted during the post-stress recovery period [52]. Fischer rats appear to be characterized by a more sustained HPA activation after exposure to acute restraint stress for 4 h or after daily repeated restraint, compared with both Lewis and SD rats [46].

In accordance with data in females, Lewis and Fischer males do not appear to differ in basal CRH gene expression in the PVN [41, 53]. We are not aware of any study about PVN CRH gene expression after acute stress in males of these strains, but chronic immobilization (IMO)-induced increase in CRH gene expression was similar in the two strains [41]. More recently, Ergang and associates [54] compared males exposed or not to a 3 days restraint stress protocol with a sacrifice at 2 h after the last stressor and studied various parameters related to central aspects of the HPA axis. Although this protocol does not allow to distinguish between the protracted consequences of prior restraint and that of the last restraint exposure, no differences between Lewis and Fischer rats were observed in PVN CRH or vasopressin gene expression.

In some studies where both males and females were simultaneously studied, a lower HPA response to forced swim, restraint or a novel environment was observed in Lewis than Fischer rats of the two sexes [43, 55, 56]. In contrast, Gomez-Serrano *et al.* [57] also studied both sexes and reported lower corticosterone response to endotoxin (at 2 h) in Lewis than Fischer females, but no differences in males. Spinedi *et al*. [42] did not observe any difference between Lewis and Fischer of the two sexes in ACTH response to insulin-induced hypoglycemia or ether but did observe a reduced response to immune stimuli (*e.g*., endotoxin), though restricted to females. That altered PVN function is more dramatic in female and male Lewis rats is supported by the finding that, in contrast to the reduced *c-fos* expression after stress observed in the PVN of Lewis *vs*. Fischer females, no differences in *c-fos* response to footshock or interleukin-1beta has been found in males [58]. In sum, the lower HPA response to the stress of male Lewis compared with male Fischer rats might not be so general as that of females.

Interestingly, to our knowledge, only one laboratory has measured CBG levels in these two strains and also in SD rats [39, 44], but they did not include females. After exposure to restraint stress, CBG levels were higher in Fischer than Lewis, but despite these higher CBG levels, free corticosterone levels were still higher in Fischer than in the other strains, with no differences between Lewis and SD. These results point again to the idea that Fischer rats are in the extreme of high HPA responsiveness to stressors.

When looking at possible overall indexes of HPA activity, results indicate a lower relative adrenal weight and higher relative thymus weight in both male and female Lewis rats compared to Fischer rats [28, 41, 46, 53, 59, 60], confirming lower HPA activity in Lewis. Although the integrity of negative GCs feedback has been assessed in only one study, it appears that Lewis and Fischer do not differ in the sensitivity of ACTH and corticosterone response to DEX in stress conditions. However, blockade of corticosterone synthesis increased ACTH in Fischer and other strains but not in Lewis [61]. This supports the hypothesis of the impaired capability of the HPA axis of Lewis to stimulatory inputs rather than enhanced negative feedback. In fact, no differences in GR or MR expression in the hippocampal formation have been found between the two strains [41].

To demonstrate that Lewis and Fischer are likely to be in the extreme of differential HPA responsiveness, comparisons with other strains are very useful. Both male and female Lewis rats showed lower ACTH and corticosterone responses to forced swimming than Fischer, but also than other inbred strains such as Brown-Norway, spontaneously hypertensive (SHR) and Wistar Kyoto (WKY) [43, 45]. The impaired ACTH and corticosterone response to stress (novel environment) of female Lewis rats is also observed compared with two other strains (Brown-Norway and Dark Agouti) [62]. In males, it has been reported a reduced ACTH response to various stressors (tail-cut and sampling, open-field, IL-1β, ether-surgery), with low or null differences in corticosterone [63]. In addition, reduced CRH gene expression in the PVN under basal conditions and impaired *in vitro* adrenocortical responsiveness to ACTH were found, also suggesting defective adrenocortical function. When compared with SD rats, Lewis also showed reduced ACTH [64] and corticosterone responses [46, 47, 65] to restraint. Reduced corticosterone response to amphetamine was also observed [65]. The reduced HPA response to restraint in Lewis compared with SD is compatible with a reduced *c-fos* response in the PVN [66]. In the latter study, strain differences in *c-fos* expression were dependent on the particular brain area, with a reduced response being observed only in a subset of the areas studied, for instance, the PVN and the medial prefrontal cortex, whereas in others (locus coeruleus) even a higher response was found.

The above data indicate that defective HPA function is a characteristic of Lewis rats as compared with some other rat strains and not only with Fischer. However, some studies also suggest that Fischer rats might be characterized by certain HPA hyperactivity as compared with other inbred rat strains [43, 67]. Comparing male Fischer and SD, the former showed higher corticosterone response to restraint, but differences were greater after chronic restraint, suggesting also impaired adaptation [39, 44, 46, 68].

## 3 HPA ACTIVITY IN STRAINS OR LINES DIFFERING IN EMOTIONALITY AND COPING

### 3.1 Maudsley Rats

Maudsley reactive and non-reactive (MR, MNR) strains were selected on the basis of emotional reactivity (mainly defecation rate) to open-field exposure, the MRN being the one most deviating from the outbred population [69, 70]. The two strains differ in other behaviors, and enhanced anxiety (elevated plus maze, EPM) and immobility (forced swim) have been observed in MR [71] associated with lower acoustic startle response [72]. The two strains do not differ either in basal levels of corticosterone or their responsiveness to an open-field, restraint or forced swim [73, 74]. More recently, the lack of differences between the two strains was confirmed by measuring both ACTH and corticosterone responses to open-field or inescapable foot-shocks [75]. Interestingly, all the above results were obtained from colonies maintained in the USA. In the only study using MR and MNR rats bred outside the USA (*i.e*., from the Queen Mary Univ. London), greater ACTH but normal corticosterone response to 30 min restraint was observed in MR than MNR rats [76]. The reasons underlying these differences are unclear, but genetic drift or animal facility breeding differences could contribute.

### 3.2 High and Low Anxiety-related behavior Lines (HAB, LAB)

HAB and LAB rats were selected from Wistar on the basis of open arm avoidance in EPM and this also resulted in HAB rats being less active in novel environments and more passive in the forced swim test (FST) [77]. This strongly suggests that HAB rats are both more anxious and prone to adopt passive strategies than LAB rats. Interestingly, whereas no ACTH or corticosterone differences were found in response to the EPM or the FST [77], greater HPA and prolactin responses were observed after forced exposure to the open arms of an EPM [78], suggesting that it is forced exposure that caused to the open space that caused the greater response. In a further study, greater ACTH and corticosterone levels in response to CRH in DEX-treated rats (DEX+CRH test) were found in HAB together with greater vasopressin PVN expression [79]. However, in response to social defeat, greater rather than lower HPA response was found in LAB *vs*. HAB rats [80]. These findings are of major relevance as they illustrate two major points: first, anxiety is not necessarily related to increased HPA responsiveness to stressors; and, second, exposure to different types of emotional stressors is important when characterizing individual/strain differences.

### 3.3 Performance in Two-way Active Avoidance Tasks (TWAA)

#### 3.3.1 Syracuse Rats

Syracuse high and low avoidance (SHA, SLA) rats originated from Long-Evans rats and differ not only in TWAA, but also in other behaviors: SLA showed normal open-field activity, improved passive avoidance and greater conditioned emotional response [81-83]. SLA showed higher adrenal weight and adrenal cortex and medulla size [84-86]. In both sexes, SLA showed higher adrenal and probably higher basal corticosterone levels but a similar response to ether exposure (although adrenal content was higher in SHA) [85, 83]. Given that only the response to a high-intensity systemic stressor was studied and that ACTH response was not measured, no firm conclusions can be drawn, although greater HPA activity associated with low avoidance cannot be disregarded.

#### 3.3.2 Hatano Rats

Hatano high and low avoidance rats (HAA, LAA) derived from SD [87]. The LAA rats of both sexes have very poor avoidance performance that does not improve over the days and HAA rats, in addition to a good performance in the TWAA task, are much more active in a running-wheel and an open-field [87, 88]. In both sexes, the adrenal weight was found to be higher in HAA than LAA and the ACTH response to the task was also clearly higher in HAA, with no differences in corticosterone [89]. In a further study, ACTH response to another emotional stressor (restraint) was markedly higher in HAA males, whereas changes in corticosterone were complex but not different overall [90]. In the latter study, the higher ACTH response was not a reflection of generalized neuroendocrine hyperresponsiveness, as prolactin response to restraint was lower in HAA. However, whereas the data regarding HPA response to restraint was further replicated, no differences in prolactin were found [91]. In the most recent study with these strains, the neuroendocrine response to the first and the last of three TWAA sessions was assessed [92]. HAA showed greater ACTH but lower corticosterone responses on days 1 and 3. The greater ACTH response was consistent with previous studies, but the lower corticosterone response was not. If consistent, data might be suggestive of impaired adrenocortical responsiveness to ACTH in HAA rats despite their higher adrenal weight.

#### 3.3.3 Roman High and Low Avoidance Rats

Roman high and low avoidance (RHA, RLA) lines were generated by Bignami [93] in Italy on the basis of performance in TWAA. It has been later demonstrated that these lines also differ in several behavioral characteristics, RLA being more anxious and emotional and more prone to adopt passive coping strategies [94]. Characterization of the neuroendocrine response to stress in these lines was first conducted in animals maintained in Driscoll’s lab in Switzerland. In a seminal and elegantly designed study, Gentsch *et al*. [27] showed that RLA and RHA lines did not differ in basal levels of classical stress markers (ACTH, corticosterone, prolactin or glucose). However, RLA showed a greater endocrine response when exposed to relatively mild stressors (saline injection, novel cage, open-field) but not to more severe stressors such as ether, immobilization or inescapable foot-shocks (except greater prolactin levels after foot-shocks). In contrast, no differences in the glucose response to any stressor were found. A further study confirmed the greater HPA response of RLA rats to mild stressors in addition to a greater *in vivo* ACTH response to exogenous CRH [95]. However, relative adrenal weight has given inconsistent results [96, 95]. In sublines derived from Switzerland stock but bred in Bordeaux, higher HPA response to an open-field in RLA was only observed at certain ages, whereas consistently higher prolactin response was found at all ages [97]. It thus appears that the greater stress sensitivity of RLA rats is better observed with prolactin. Interestingly, neonatal handling decreased HPA and prolactin stress responsiveness of RLA rats and did not affect RHA rats, thus eliminating the typical higher neuroendocrine responsiveness of RLA rats [98].

Are there differences in the HPA axis at the level of the PVN? Enhanced corticosterone response to an open-field in RLA was associated with normal CRH gene expression but enhanced vasopressin expression in the mpdPVN [99]. This is of interest as vasopressin gene expression in the mpdPVN typically increases when the HPA axis is chronically more active [12]. Taking advantage of the inbreeding process carried out in these lines, we performed a characterization of the HPA axis in RHA-I and RLA-I rats [100]. We found no differences in basal levels of ACTH or corticosterone, but a higher HPA response to mild stressors in RLA rats. In addition, we also detected a higher *Crh* gene expression in the PVN, suggestive of a more active HPA axis, with no differences in adrenal weight or the expression of corticosteroid receptors (GR or MR) in critical brain regions, including the PVN. A further study in these inbred animals has confirmed the higher HPA and prolactin responses to a novel environment of RLA *versus* RHA rats and showed that the pattern of the former rats was similar to that of genetically heterogeneous stock [101]. Overall, the data suggest lower neuroendocrine responsiveness to stress in RHA rats.

### 3.4 HPA Activity in Rat Strain/Lines Showing Depression-like Behavior

#### 3.4.1 Wistar Kyoto Rats

WKY and SHR are derived from Wistar rat ancestor in Kyoto, but they have been obtained by two independent inbreeding processes [102, 103]. Interest was initially focused on SHR as a putative animal model of essential hypertension and some studies in this regard have compared SHR with WKY, whereas other studies compared SHR with outbred Wistar or other strains. From a behavioral perspective, they are of interest as putative animal models of attention deficit and hyperactivity disorder (SHR) [104] and depression (WKY) [105]. WKY rats were initially characterized by extreme passive behavior in the FST and for being resistant to antidepressants [106-108].

There is ample evidence for enhanced sympathetic activity and stress-induced catecholamine release under stress conditions in SHR compared with WKY and other normotensive rat strains [109] and this will not be discussed here. Importantly, when SHR and WKY have been studied together with WK-HA (hyperactive) and WK-HT (hypertensive), greater plasma catecholamine response to stress is associated with hyperactivity and not hypertension [110]. In the present review, we will focus on alterations in the HPA axis in WKY as compared with SHR and other strains [110]. Since most studies comparing WKY and SHR have been done in males, specific references to sex will be made only when females or both sexes were used.

Inconsistent results have been reported regarding relative adrenal weight, with greater [111-115] or similar [116-120] weight in male SHR compared with WKY. In a study with both sexes and three different ages in adult rats, no differences were generally observed in adrenal weight, whereas, in contrast, consistently reduced thymus weight was found in SHR [121] suggesting hyperactivity of the HPA axis in SHR *versus* WKY. However, when WKY are compared with Brown-Norway or SD rats, there is no evidence of lower adrenal weight in WKY [122, 123].

Male WKY compared with SHR have been shown to have lower [124-126], similar [43, 41, 61, 127] or higher [128] basal levels of corticosterone release. Hashimoto *et al*. [126] showed lower corticosterone levels in male WKY despite similar ACTH levels. Age might contribute to discrepancies as similar basal corticosterone levels were observed in WKY and SHR between 4 and 12 weeks and transiently higher levels at 16 weeks in WKY that normalized again at 20 weeks [129].

CRH gene expression in the PVN has been found to be either similar in WKY and SHR [41] or lower in WKY and Wistar compared with SHR [130]. Nevertheless, differences were more evident in the latter study after exposure to acute restraint stress. No differences in POMC gene expression in the anterior pituitary have been observed in one study [131], whereas in another POMC gene expression was higher in WKY than SHR [132]. It seems that available studies did not offer a clear picture of the HPA function at anterior pituitary and PVN levels.

Results are also controversial regarding the response to stressors. After ether exposure, the corticosterone response of WKY has been shown to be higher [128], similar [119] or lower [133] compared with SHR. In the latter study, WKY, SHR and outbred Wistar rats were included and lower ACTH and corticosterone responses of both WKY and Wistar *versus* SHR were found, with similar responses in WKY and Wistar at all ages. Interestingly, differences were more marked in very young rats and progressively vanished, with no differences in 16-week-old rats [133]. These results are suggestive of a critical contribution of age with the higher responsiveness of SHR with respect to WKY and other strains being better observed at young ages. However, Hashimoto *et al*. [126] observed higher ACTH and corticosterone responses to ether or hemorrhage in 7-week-old WKY than SHR, associated with a lower corticosterone response. This higher ACTH response was accompanied by a lower ACTH responsiveness to exogenous CRH and a similar response to exogenous vasopressin, suggesting that differences might be related to higher stress-induced CRH release but lower adrenal responsiveness to ACTH in WKY than SHR. Although the results are puzzling, they illustrate the importance of measuring both ACTH and corticosterone and checking the responsiveness of the corticotrope cells and the adrenal cortex.

The picture is not clearer with more emotional stressors. After immobilization, similar corticosterone levels were found in WKY and SHR in one study [124] and lower levels in WKY in another [134]. A lower corticosterone response was also observed in WKY after a brief exposure to foot-shocks [135]. However, the corticosterone response elicited by daily sessions of TWAA involving foot-shocks did not differ between the two strains [136]. A similar ACTH response to forced swim (both sexes) and tail-shocks (males) has been observed [43, 61], although corticosterone levels were higher in males but not females [43]. This suggests enhanced adrenal responsiveness in WKY males, which is in contrast to Hashimoto *et al*. [126]. Regarding the response of the two strains to exogenous DEX administration, a similar reduction of the HPA response to tail-shocks was found [61], with no evidence of altered negative GCs feedback.

In sum, the comparison of the activity of the HPA axis in WKY and SHR has resulted in a complex picture, with no consistent overall differences in contrast to the well-characterized hyperreactivity of catecholamines. The characterization of the HPA function in WKY compared with strains other than SHR can give us a clearer picture of the putative particularities of WKY.

When the circadian ACTH and corticosterone patterns were compared in WKY and Wistar, similar levels were found during lights on, but during the lights off, greater ACTH and corticosterone levels were found in WKY [137]. Accordingly, higher POMC expression in the anterior pituitary was observed in WKY *versus* Wistar [138]. In the same study, *in vitro* basal ACTH release was higher in WKY, but the response to CRH and vasopressin was lower, consonant with lower CRHR1 binding and expression in the anterior pituitary. All these data suggest that corticotrope cells of WKY might have higher intrinsic activity but a lower response to hypothalamic inputs than Wistar. Unfortunately, HPA response to stress was not assessed in the same study, but a higher ACTH response to cold-restraint stress has been reported in WKY compared with Wistar, associated with a similar corticosterone response [137]. PVN CRH gene expression does not appear to be different from some other inbred strains [41] or from FIS and SD rats [139], but in another study higher expression was found *versus* SD [140]. In accordance with the latter results, WKY showed similar basal levels of ACTH as SD and Lewis, but greater ACTH levels than SD during the recovery period after being exposed to 60 min restraint, whereas Lewis showed, as expected, a lower response than the other two strains [64]. In the same line, similar basal levels but greater ACTH and corticosterone responses to forced swim were found in WKY than in SD [141]. Of note, a study by Redei *et al*. [142] compared WKY with Fischer and Wistar after manipulating corticosterone levels by adrenalectomy with or without exogenous corticosterone supplementation (Sham, ADX, ADX+C), in order to study the contribution of corticosterone to water-restraint stress-induced ulcers. Lower corticosterone levels were found in ADX+C WKY than Fischer and Wistar, suggesting enhanced steroid metabolism. This is an aspect that has not been basically studied and can contribute to explain discrepancies between ACTH and corticosterone.

To our knowledge, the responsiveness of WKY to acute GCs manipulations has been tested in only two studies [142]. In the first study, no differences in ACTH and corticosterone response to tail-shocks or in the response to DEX-induced negative feedback or pharmacological adrenalectomy were found in WKY compared with SHR and Fischer [61]. In the second study, no differences in DEX-induced suppression of basal ACTH were found between WKY and SD, whereas impaired suppression was apparent with corticosterone [141], which could be explained by enhanced corticosterone metabolism.

In addition to the few studies involving both sexes, only two additional studies have explored differences in HPA hormones in WKY and SHR females, both under basal conditions. A study compared female WKY and SHR together with SD and no differences in corticosterone were found [143]. In adolescent females, corticosterone levels of WKY were lower than that of SHR [144], suggesting a contribution of age to the strain differences, as reported in males.

#### 3.4.2 Flinders Sensitive and Resistant Lines

Flinders sensitive and resistant lines (FSL, FRL) were genetically selected on the basis of the response to muscarinic drugs and were found to differ in coping behavior in the FST and other behaviors, with FSL showing depression-like characteristics [145]. Some studies have compared both lines and others FSL with SD as controls. Baseline levels of corticosterone did not differ between FRL and FSL, but the response to the muscarinic cholinergic agonist arecoline was higher in FSL [146], in accordance with the criteria used for genetic selection. Later, Owens *et al*. [147] observed lower basal ACTH but normal corticosterone in FSL. However, no differences in basal corticosterone levels were reported between FRL and FSL either in unstressed controls or after exposure to chronic unpredictable stress (CUS), despite the expected increase in basal corticosterone after CUS [148]. In response to noise stress, reduced corticosterone was found in FSL than FRL, whereas similar corticosterone levels were observed in the two lines after restraint or colorectal distension under restraint [149]. Although ACTH levels were not measured, the lower corticosterone response to a mild stressor is suggestive of a reduced HPA responsiveness to stress in FSL *versus* FRL. Urinary excretion of corticosterone during 24 h exposure to metabolic cages is similar in the two lines [150].

In some other studies, FSL have been compared with typical strains (*e.g*. SD) and the results are puzzling. Inconsistencies were observed within the same laboratory [151, 152]: presumably basal levels of ACTH and corticosterone were lower in FSL in one study [151], whereas in another ACTH did not differ and corticosterone levels were higher in FSL [152]. In pre-weanling rats, FSL showed slightly higher ACTH response to exposure to a novel cage or an adult rat, but corticosterone response was similar [143, 144, 153]. Thiele *et al*. [154] observed higher basal corticosterone levels in males FSL than in males SD and the same trend in females, whereas in another study with males, no differences were observed [155]. However, Thiele *et al*. [154] compared FSL rats bred in their center with SD rats purchased from Charles-River, which is in general non-appropriate. Finally, Krokas *et al*. [156] studied corticosterone levels in male and female FSL and SD rats 20 min after behavioral testing (open-field and EPM) on the day after chronic vehicle or citalopram administration: higher corticosterone levels were observed in male FSL *versus* SD vehicle-treated rats that disappeared in citalopram-injected rats, whereas no difference was observed in females. The origin of the two strains was not reported.

### 3.5 Rats Genetically Selected for Stress Corticosterone Responsiveness

It is quite surprising that genetic selection of low or high HPA responsiveness to stressors has only recently been approached by Sandi’s lab. They exposed young rats for three days (postnatal days 28-30) to two relatively mild stressors daily and used plasma corticosterone on the last day to classify rats in low, intermediate and high responders (LR, IR and HR, respectively). Several males and females from each group were crossed and exposed to the same procedure over generations to establish the three lines. Lines did not differ in relative adrenal weight or basal corticosterone levels (at nadir or peak of the circadian rhythm), but did in corticosterone response to restraint stress [157]. Lines also differed in cardiovascular regulation with higher heart rate and basal vagal tone in both LR and HR compared with IR [158]. HR rats appear to be slightly more anxious (EPM) and prone to adopt passive strategies in the FST than LR, but showed clearly higher offensive behavior [159]. In addition, the impact of juvenile stress exposure was quite similar, except that offensive behavior was increased in LR but not HR rats. Differences have also been observed regarding strategies used for spatial learning (Morris water maze), with overall higher long-term memory in HR [160]. Whether physiological and behavioral differences between lines are causally linked to altered corticosterone responsiveness to stress remains to be studied. Nevertheless, in order to delve into the contribution of constitutive differences in the HPA axis on behavior and the consequences of exposure to stress, it would be of great interest to develop other lines differing in HPA axis activity.

## 4 OVERALL DISCUSSION

In-depth evaluation of strain/lines differences in the HPA is scarce. Essential aspects to consider are the characterization of the circadian pattern of corticosterone, the measurement of stress levels of both ACTH and corticosterone and the report of the relative weights of adrenal glands and thymus, which can provide us with important complementary information.

### 4.1 Methodological Concerns

Although individual studies characterizing the HPA axis in different rat strains/lines are necessarily limited in scope, a point of major relevance in any study is whether obvious methodological concerns are detected that can lead to misinterpretation of data. In addition to the difficulties in reporting true basal levels of ACTH and corticosterone, probably the most common drawback of the available literature data, a major concern when comparing inbred strains is whether or not they can be obtained from the same breeding center. Important details that might differ between centers and hence affect the results are the number of pups per mother, the time of weaning, the characteristics of the home cages (and possible enrichment) and the degree of perturbations in the animal rooms by laboratory routines. A second major concern is that most published results have characterized strain differences only in males and data in females are scarce. The exception to this rule is the Lewis and Fischer rats, in which earlier studies were done in females and many publications are available with both sexes.

### 4.2 Answers to Critical Questions on the Basis of Experimental Evidence

At present, there is no evidence for a genetic selection resulting in high overall reactivity to emotional stressors deduced from the responsiveness of several classical neuroendocrine stress systems. Classical physiological markers of stressor intensity included plasma levels of ACTH (and corticosterone with some limitations), catecholamines (adrenaline, noradrenaline), glucose, as a surrogate of adrenaline release, and prolactin [5]. In most cases, higher responsiveness of a particular strain/line is restricted to one or two of them. Table **1** summarizes the results obtained comparing stress-sensitive variables other than ACTH and corticosterone in some relevant rat strains.

The best example of a dissociation between different stress markers is the pair SHR-WKY. Whereas consistent hyperreactivity to stress in terms of adrenaline and noradrenaline is found in SHR [124, 134], differences in the reactivity of the HPA axis are controversial and no differences in the prolactin response to stress have been found [43]. A second example is the Lewis-Fischer pair, in which the defective HPA response to stress detected in Lewis was not observed with prolactin [43]. In the only available study regarding plasma catecholamines, a lower response to IMO was found in Lewis compared with Fischer [161], suggesting parallelism with the HPA axis.

To our knowledge, the most marked differences in the HPA axis are found in outbred Long-Evans rats as compared with some other outbred or inbred strains, including Fischer [168]. Long-Evans also appear to have a greater prolactin response to stressors than Wistar [169] and higher levels of enzymes involved in catecholamine synthesis in the adrenal compared with SD, suggestive of enhanced CA responsiveness. When considering another pair of strains widely studied, Fischer and Lewis, clearly lower levels of ACTH after acute restraint were observed in Lewis, in parallel with modest but significantly lower plasma adrenaline responsiveness [170]. However, no differences between Lewis and SD were found in the prolactin response to IMO despite the expected lower corticosterone response in Lewis [65].

Is HPA responsiveness independent of the type of emotional stressor? This is a question difficult to answer as most studies focused only on one single stressor. However, it is likely that the type of stressor is important. In an outbred population of rats, individual differences are consistent when stressors are of similar intensity (exposure to novel environments) but are not when comparing stressors greatly different in intensity (*e.g*., immobilization *versus* novel environment) [171-173]. If this applies to genetically-selected animals, particular characteristics of stressors might be relevant and should be tested when describing the stress phenotype of particular rat strains. Some data supporting an important role of the type of stressor follows. First, in RHA-RLA rats, higher ACTH and prolactin responses were observed in RLA in response to relatively mild stressors but not to more severe stressors [96]. Second, in HAB-LAB rats, higher ACTH and prolactin responses were observed in HAB after forced exposure to the open arms of the EPM but not after free exploration of the EPM, and social defeat resulted in lower ACTH response in the more anxious HAB rats (see above).

### 4.3 Is HPA Response to Stress Related to Anxiety?

The results obtained with HAB-LAB rats nicely demonstrated that there is no obvious relationship between anxiety-like characteristics derived from the EPM and HPA responsiveness to stressors. These data are in accordance with our data in outbred rats [171]. Interestingly, differences between the two lines were dependent on the type of emotional stressor, introducing major concerns regarding simple characterization of trait-like differences in HPA responsiveness. Thus, forced open arm exposure did result in a greater hormonal response in HAB, suggesting specific fear to open areas, rather than generalized stress hyperresponsiveness. Perhaps even in outbred rats, those showing lower open arm entries during EPM exposure will show higher response with forced exposure.

Interpretation of good *versus* poor performance in the TWAA is difficult because of the contribution of several factors. Evidence obtained in RHA-RLA rats has demonstrated a major contribution of anxiety to impair TWAA acquisition but also of coping style, with passive coping predisposing to freezing, which in turn is detrimental to engaging in the active behavior needed for avoidance. Since in these rats, RHA showed greater neuroendocrine responsiveness to stressors (at least when they are of relatively low intensity), high anxiety and passive coping appear to be associated with increased neuroendocrine stress responsiveness. What is the conclusion derived from Hatano lines? These rats were selected by TWAA performance, but those rats showing higher levels of freezing were eliminated during the process of genetic selection [89]. In these rats, HAA appears to be more anxious than LAA rats, this increased anxiety contributing to their better avoidance performance. HAA also showed a greater HPA response to stress, the data favoring a positive relationship between anxiety and HPA responsiveness, at least within animals that are prone to active coping. This might suggest that enhanced HPA responsiveness might be a characteristic of high anxiety, regardless of coping style. However, it is possible that high-anxiety passive copers could manifest enhanced HPA responsiveness to mild stressors whereas enhanced HPA responsiveness would still be observed after exposure to more severe stressors in high-anxiety active copers.

### 4.4 FST and Coping

Behavior in the FST does not appear to be related to anxiety and might instead reflect coping style [174]. A critical question is whether passive coping in the TWAA task (*i.e*., freezing) is related to passive coping in the FST. Although correlation studies have not been done, results in RHA-RLA and NIH rats suggest a parallelism between freezing behavior in the TWAA context and immobility during the first 5 min of the FST [101].

The HPA axis does not appear to be related to coping behavior in the FST. This was the main conclusion achieved by comparing several different inbred rat strains [43, 45]. This is nicely supported by Redei and collaborators' studies with WKY sub-strain differing in immobility in the FST [175, 176]. Since WKY does not appear to be completely inbred, the authors selectively bred WKY for low and high immobility in the FST, but basal or restraint stress corticosterone levels were similar in the two substrains [175]. A similar conclusion was reached using F2 of WKYxFIS, concluding that depressive-like behavior in the FST and HPA function (basal or stress corticosterone and adrenal weight) were dissociated [176].

### 4.5 Guide to Explore Individual or Strain Differences in the HPA

There appears to be a general agreement about the importance of characterizing individual differences in critical physiological and behavioral traits. Comparison of outbred populations of rodents is a good approach, but exploitation of available outbred and inbred strains could allow us to establish or rule out important relationships between different aspects of behavior or between relevant physiological aspects and behavior. However, advances in the field also require one to be aware of methodological problems and reject excessively simple explanations. We have summarized in Tables **2** and **3** both methodological considerations and their implications as well as a recommended guide to better interpret experimental data about the HPA axis. We hope this could be of value to those interested in this relevant endocrine system.

## CONCLUSION

The activity of the HPA axis and its main output, GCs, is considered to be critical for coping and adaptation to stress and it has been associated with a number of psychiatric diseases, including anxiety and depression. Consequently, attention has been devoted to the characterization of the HPA function in rodent strains differing in particular physiological or behavioral aspects. However, the eventual consequences of the described differences are unclear. The present review of selected rat strains shows that previous approaches on this subject have been incomplete and plagued by methodological problems. Therefore, available data are very often controversial. Nevertheless, the overview of all these data strongly suggests that there is no simple relationship between HPA activity and anxiety-like behavior, depression-like behavior and coping style. We need new perspectives about the putative role of the HPA axis in these extremely relevant traits and neuropsychiatric diseases.

## Figures and Tables

**Fig. (1):**
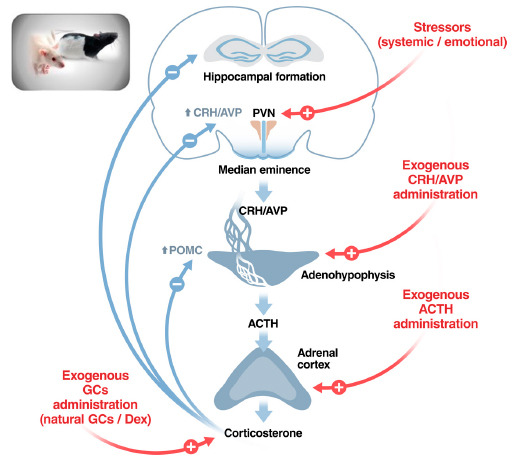
A schematic view of the HPA axis and how to test functional strain differences. Possible loci of strain differences in the regulation of the HPA axis, an example of a complex neuroendocrine system. Differences might exist under resting conditions that are likely to be reflected also in response to stressors, but an altered response to stressors or other stimuli might exist in the absence of differences under resting conditions. Main stimuli used to test differences in the HPA axis are indicated in red. **Abbreviations:** ACTH: Adrenocorticotropic hormone; AVP: Vasopressin; CRH: Corticotropin-releasing hormone; DEX: Dexamethasone; GCs: Glucocorticoids; POMC: Proopiomelanocortin (precursor of ACTH); PVN: Paraventricular nucleus of the hypothalamus. Figure designed by the co-author Xavier Belda.

**Table 1:** Comparison of resting and stress levels of biological parameters in selected strain pairs.

**Strain Pairs**	**Plasma Catecholamines**		**Glucose**		**Prolactin**	
	**Basal**	**Stress**	**Basal**	**Stress**	**Basal**	**Stress**
Lewis-Fischer	E ↓ NE = [161]	E ↓ NE ↓ [161]	= [43]	↓ [43]	= [43]	= [43]
Lewis-SD	E ↓ NE = [162]	E ↓ NE ↓ [162]	ND	ND	= [65]	= [65]
HAB-LAB	ND	ND	ND	ND	↑ [78]	↑ [78]
RLA-RHA	ND	ND	= [27,163]	= [27]	= [27,97,101,164,165]	↑ [27,97,98,101,164,165]
WKY-SHR	See [109]	See [109]	= [43]	↓ [43]	= [43,166], ↑ [167]	= [43]
FSL-FRL	ND	ND	ND	ND	= [150]	↑ [150]

**Note:** Differences are indicated by symbols, always using as the reference the first strain or the pair. **Abbreviation:** ND = not determined.

**Table 2:** Methodological concerns.

Consideration	Implications
In rats and mice, resting levels of corticosterone in the morning hours are about 10-20 ng/ml (1-2 µg/dl) when measured by radioimmunoassay (RIA) or enzyme-immunoassay (ELISA). Most published studies present values between 2- and 10-fold higher.	Values usually reported in published studies are indicative of stress associated with blood sampling and do not reflect true HPA resting levels.
Under certain conditions (circadian rhythm, acute stress prolonged for several hours, chronic stress) corticosterone levels do not reflect ACTH levels.	If we consider ACTH the primary response to a stressor, measuring only corticosterone can lead to erroneous interpretations.
Strain or individual differences in the HPA response might differ between systemic and emotional stressors.	Results cannot be extrapolated from one type of stressor to another.
Genetic selection could have resulted in altered responsiveness of one particular system rather than general emotional reactivity to stressors.	Strain differences in the HPA response to emotional stressors are not necessarily indicative of a more general endocrine response.

**Table 3:** Looking for the main locus of individual/strain differences in stress responsiveness.

Aspects to be Considered	Observations
1. Animals differ in a general construct of emotional reactivity	If strains differ in emotional reactivity, differences are expected to be in the same direction in all or most of the physiological responses that are sensitivity to the intensity of stressors. If this is true, differences should be observed in the physiological response to emotional but not systemic stressors.
2. Animals differ in the activity of a particular physiological system	Genetic selection affects a wide range of genes related to different functions. If the selected genes have impact on a particular physiological system (*e.g*., HPA axis), we can observe differences in this particular system but not in other stress-related systems (*e.g*., prolactin). We cannot infer from a particular system that the two strains differ in responsiveness to stress, as the differences are likely to be restricted to a particular system.
3. Which is the locus of differences in a particular system?	Suppose we detect differences in corticosterone response to stress between two strains. As discussed previously, the critical locus might be at different levels of the HPA axis: a) Processing of inputs arriving at the PVN and the corresponding release of CRH and other ACTH secretagogues. b) Responsiveness of corticotrope cells to hypothalamic stimulatory factors. c) Responsiveness of adrenocortical cells to ACTH. d) Altered corticosterone metabolism. e) Altered sensitivity to negative glucocorticoid feedback at any level.
4. Sensitivity of tissues to circulating corticosterone	This can be linked to changes in corticosteroid receptor expression or to other independent cell characteristics. Differences cannot be extrapolated from one cell or tissue to others

## LIST OF ABBREVIATIONS

CBG: Corticosteroid-binding Globulin
FST: Forced Swim Test
GCs: Glucocorticoids
HPA: Hypothalamic-pituitary-adrenal
MDR1: Multidrug-resistant Protein 1
RHA: Roman-high Avoidance
RIA: Radioimmunoassay
SD: Sprague-dawley
SHR: Spontaneously Hypertensive

## References

[1] Vigas M., Usdin E., Kvetnansky R., Kopin I. (1980). Contribution to the Understanding of the Stress Concept.. Catecholamines and stress: Recent advances..

[2] Herman J.P., McKlveen J.M., Ghosal S., Kopp B., Wulsin A., Makinson R., Scheimann J., Myers B. (2016). Regulation of the hypothalamic- pituitary-adrenocortical stress response.. Compr. Physiol..

[3] Sapolsky R.M., Romero L.M., Munck A.U. (2000). How do glucocorticoids influence stress responses? Integrating permissive, suppressive, stimulatory, and preparative actions.. Endocr. Rev..

[4] Finsterwald C., Alberini C.M. (2014). Stress and glucocorticoid receptordependent mechanisms in long-term memory: From adaptive responses to psychopathologies.. Neurobiol. Learn. Mem..

[5] Armario A., Labad J., Nadal R. (2020). Focusing attention on biological markers of acute stressor intensity: Empirical evidence and limitations.. Neurosci. Biobehav. Rev..

[6] Chrousos G.P., Gold P.W. (1992). The concepts of stress and stress system disorders. Overview of physical and behavioral homeostasis.. JAMA.

[7] Chrousos G.P. (2009). Stress and disorders of the stress system.. Nat. Rev. Endocrinol..

[8] Lovallo W.R. (2011). Do low levels of stress reactivity signal poor states of health?. Biol. Psychol..

[9] Jacobson L. (2014). Hypothalamic-pituitary-adrenocortical axis: Neuropsychiatric aspects.. Compr. Physiol..

[10] Armario A. (2006). The hypothalamic-pituitary-adrenal axis: What can it tell us about stressors?. CNS Neurol. Disord. Drug Targets.

[11] Spencer R.L., Deak T. (2017). A users guide to HPA axis research.. Physiol. Behav..

[12] Aguilera G., Rabadan-Diehl C. (2000). Vasopressinergic regulation of the hypothalamic-pituitary-adrenal axis: Implications for stress adaptation.. Regul. Pept..

[13] Bornstein S.R., Engeland W.C., Ehrhart-Bornstein M., Herman J.P. (2008). Dissociation of ACTH and glucocorticoids.. Trends Endocrinol. Metab..

[14] Keller-Wood M. (2015). Hypothalamic pituitary - Adrenal axis-feedback control.. Compr. Physiol..

[15] Meijer O.C., de Lange E.C.M., Breimer D.D., de Boer A.G., Workel J.O., de Kloet E.R. (1998). Penetration of dexamethasone into brain glucocorticoid targets is enhanced in mdr1A P-glycoprotein knockout mice.. Endocrinology.

[16] Mason B.L., Pariante C.M., Thomas S.A. (2008). A revised role for Pglycoprotein in the brain distribution of dexamethasone, cortisol, and corticosterone in wild-type and ABCB1A/B-deficient mice.. Endocrinology.

[17] Pruessner J.C., Wolf O.T., Hellhammer D.H., Buske-Kirschbaum A., von Auer K., Jobst S., Kaspers F., Kirschbaum C. (1997). Free cortisol levels after awakening: A reliable biological marker for the assessment of adrenocortical activity.. Life Sci..

[18] Belda X., Fuentes S., Labad J., Nadal R., Armario A. (2020). Acute exposure of rats to a severe stressor alters the circadian pattern of corticosterone and sensitizes to a novel stressor: Relationship to pre-stress individual differences in resting corticosterone levels.. Horm. Behav..

[19] Raff H., Bruder E.D., Cullinan W.E., Ziegler D.R., Cohen E.P. (2011). Effect of animal facility construction on basal hypothalamicpituitary- adrenal and renin-aldosterone activity in the rat.. Endocrinology.

[20] Döhler K.D., Gärtner K., von zur Mühlen A., Döhler U. (1977). Activation of anterior pituitary, thyroid and adrenal gland in rats after disturbance stress.. Eur. J. Endocrinol..

[21] Armario A., Lopez-Calderón A., Jolin T., Castellanos J.M. (1986). Sensitivity of anterior pituitary hormones to graded levels of psychological stress.. Life Sci..

[22] Vahl T.P., Ulrich-Lai Y.M., Ostrander M.M., Dolgas C.M., Elfers E.E., Seeley R.J., D’Alessio D.A., Herman J.P. (2005). Comparative analysis of ACTH and corticosterone sampling methods in rats.. Am. J. Physiol. Endocrinol. Metab..

[23] Pecoraro N., Ginsberg A.B., Warne J.P., Gomez F., la Fleur S.E., Dallman M.F. (2006). Diverse basal and stress-related phenotypes of Sprague Dawley rats from three vendors.. Physiol. Behav..

[24] Akana S.F., Cascio C.S., Shinsako J., Dallman M.F. (1985). Corticosterone: narrow range required for normal body and thymus weight and ACTH.. Am. J. Physiol. Regul. Integr. Comp. Physiol..

[25] Scorrano F., Carrasco J., Pastor-Ciurana J., Belda X., Rami-Bastante A., Bacci M.L., Armario A. (2015). Validation of the long‐term assessment of hypothalamic‐pituitary‐adrenal activity in rats using hair corticosterone as a biomarker.. FASEB J..

[26] Tsuchimine S., Matsuno H., O’Hashi K., Chiba S., Yoshimura A., Kunugi H., Sohya K. (2020). Comparison of physiological and behavioral responses to chronic restraint stress between C57BL/6J and BALB/c mice.. Biochem. Biophys. Res. Commun..

[27] Gentsch C., Lichtsteiner M., Driscoll P., Feer H. (1982). Differential hormonal and physiological responses to stress in Roman high- and low-avoidance rats.. Physiol. Behav..

[28] Sternberg E.M., Hill J.M., Chrousos G.P., Kamilaris T., Listwak S.J., Gold P.W., Wilder R.L. (1989). Inflammatory mediator-induced hypothalamic- pituitary-adrenal axis activation is defective in streptococcal cell wall arthritis-susceptible Lewis rats.. Proc. Natl. Acad. Sci. USA.

[29] Smith C.C., Hauser E., Renaud N.K., Leff A., Aksentijevich S., Chrousos G.P., Wilder R.L., Gold P.W., Sternberg E.M. (1992). Increased hypothalamic [3H]flunitrazepam binding in hypothalamicpituitary- adrenal axis hyporesponsive Lewis rats.. Brain Res..

[30] Sternberg E.M., Glowa J.R., Smith M.A., Cologero A.E., Listwak S.J., Aksentijevich S., Chrousos G.P., Wilder R.L., Gold P.W. (1992). Corticotropin releasing hormone related behavioral and neuroendocrine responses to stress in Lewis and Fischer rats.. Brain Res..

[31] Smith T., Hewson A.K., Quarrie L., Leonard J.P., Cuzner L. (1994). Hypothalamic PGE2 and cAMP production and adrenocortical activation following intraperitoneal endotoxin injection: In vivo microdialysis studies in Lewis and Fischer rats.. Neuroendocrinology.

[32] Calogero A.E., Sternberg E.M., Bagdy G., Smith C., Bernardini R., Aksentijevich S., Wilder R.L., Gold P.W., Chrousos G.P. (1992). Neurotransmitter-induced hypothalamic-pituitary-adrenal axis responsiveness is defective in inflammatory disease-susceptible Lewis rats: In vivo and in vitro studies suggesting globally defective hypothalamic secretion of corticotropin-releasing hormone.. Neuroendocrinology.

[33] Sternberg E.M., Young W.S., Bernardini R., Calogero A.E., Chrousos G.P., Gold P.W., Wilder R.L. (1989). A central nervous system defect in biosynthesis of corticotropin-releasing hormone is associated with susceptibility to streptococcal cell wall-induced arthritis in Lewis rats.. Proc. Natl. Acad. Sci. USA.

[34] Million M., Wang L., Martinez V., Taché Y. (2000). Differential Fos expression in the paraventricular nucleus of the hypothalamus, sacral parasympathetic nucleus and colonic motor response to water avoidance stress in Fischer and Lewis rats.. Brain Res..

[35] Patchev V.K., Mastorakos G., Brady L.S., Redwine J., Wilder R.L., Chrousos G.P. (1993). Increased arginine vasopressin secretion may participate in the enhanced susceptibility of Lewis rats to inflammatory disease.. Neuroendocrinology.

[36] Patchev V.K., Kalogeras K.T., Zelazowski P., Wilder R.L., Chrousos G.P. (1992). Increased plasma concentrations, hypothalamic content, and in vitro release of arginine vasopressin in inflammatory disease-prone, hypothalamic corticotropin-releasing hormonedeficient Lewis rats.. Endocrinology.

[37] Zelazowski P., Smith M.A., Gold P.W., Chrousos G.P., Wilder R.L., Stemberg E.M. (1992). In vitro regulation of pituitary ACTH secretion in inflammatory disease susceptible Lewis (LEW/N) and inflammatory disease resistant Fischer (F344/N) rats.. Neuroendocrinology.

[38] Grota L.J., Bienen T., Felten D.L. (1997). Corticosterone responses of adult Lewis and Fischer rats.. J. Neuroimmunol..

[39] Dhabhar F.S., McEwen B.S., Spencer R.L. (1993). Stress response, adrenal steroid receptor levels and corticosteroid-binding globulin levels a comparison between Sprague-Dawley, Fischer 344 and Lewis rats.. Brain Res..

[40] Ortiz J., DeCarpio J.L., Kosten T.A., Nestler E.J. (1995). Strainselective effects of corticosterone on locomotor sensitization to cocaine and on levels of tyrosine hydroxylase and glucocorticoid receptor in the ventral tegmental area.. Neuroscience.

[41] Gómez F., Lahmame A., de Kloet R., Armario A. (1996). Hypothalamic-pituitary-adrenal response to chronic stress in five inbred rat strains: differential responses are mainly located at the adrenocortical level.. Neuroendocrinology.

[42] Spinedi E., Salas M., Chisari A., Perone M., Carino M., Gaillard R.C. (1994). Sex differences in the hypothalamo-pituitary-adrenal axis response to inflammatory and neuroendocrine stressors. Evidence for a pituitary defect in the autoimmune disease-susceptible female Lewis rat.. Neuroendocrinology.

[43] Armario A., Gavaldà A., Mart J. (1995). Comparison of the behavioural and endocrine response to forced swimming stress in five inbred strains of rats.. Psychoneuroendocrinology.

[44] Dhabhar F., Miller A.H., McEwen B.S., Spencer R.L. (1995). Differential activation of adrenal steroid receptors in neural and immune tissues of Sprague Dawley, Fischer 344, and Lewis rats.. J. Neuroimmunol..

[45] Marti J., Armario A. (1996). Forced swimming behavior is not related to the corticosterone levels ain the test: A study with four inbred rat strains.. Physiol. Behav..

[46] Dhabhar F.S., McEwen B.S., Spencer R.L. (1997). Adaptation to prolonged or repeated stress--comparison between rat strains showing intrinsic differences in reactivity to acute stress.. Neuroendocrinology.

[47] Neeley E.W., Berger R., Koenig J.I., Leonard S. (2011). Strain dependent effects of prenatal stress on gene expression in the rat hippocampus.. Physiol. Behav..

[48] Chaouloff F., Kulikov A., Sarrieau A., Castanon N., Mormède P. (1995). Male Fischer 344 and Lewis rats display differences in locomotor reactivity, but not in anxiety-related behaviours: Relationship with the hippocampal serotonergic system.. Brain Res..

[49] Michaud D.S., McLean J., Keith S.E., Ferrarotto C., Hayley S., Khan S.A., Anisman H., Merali Z. (2003). Differential impact of audiogenic stressors on Lewis and Fischer rats: Behavioral, neurochemical, and endocrine variations.. Neuropsychopharmacology.

[50] Baumann M.H., Elmer G.I., Goldberg S.R., Ambrosio E. (2000). Differential neuroendocrine responsiveness to morphine in Lewis, Fischer 344, and ACI inbred rats.. Brain Res..

[51] Kusnecov A.W., Shurin M.R., Armfield A., Litz J., Wood P., Zhou D., Rabin B.S. (1995). Suppression of lymphocyte mitogenesis in different rat strains exposed to footshock during early diurnal and nocturnal time periods.. Psychoneuroendocrinology.

[52] Jongen-Rêlo A.L., Pothuizen H.H.J., Feldon J., Pryce C.R. (2002). Comparison of central corticosteroid receptor expression in male Lewis and Fischer rats.. Brain Res..

[53] Duclos M., Bouchet M., Vettier A., Richard D. (2005). Genetic differences in hypothalamic-pituitary-adrenal axis activity and food restriction- induced hyperactivity in three inbred strains of rats.. J. Neuroendocrinol..

[54] Ergang P., Vodička M., Soták M., Klusoňová P., Behuliak M., Řeháková L., Zach P., Pácha J. (2015). Differential impact of stress on hypothalamic-pituitary-adrenal axis: Gene expression changes in Lewis and Fisher rats.. Psychoneuroendocrinology.

[55] Stöhr T., Szuran T., Welzl H., Pliska V., Feldon J., Pryce C.R. (2000). Lewis/Fischer rat strain differences in endocrine and behavioural responses to environmental challenge.. Pharmacol. Biochem. Behav..

[56] Page G.G., Opp M.R., Kozachik S.L. (2014). Reduced sleep, stress responsivity, and female sex contribute to persistent inflammationinduced mechanical hypersensitivity in rats.. Brain Behav. Immun..

[57] Gomez-Serrano M., Tonelli L., Listwak S., Sternberg E., Riley A.L. (2001). Effects of cross fostering on open-field behavior, acoustic startle, lipopolysaccharide-induced corticosterone release, and body weight in Lewis and Fischer rats.. Behav. Genet..

[58] Rivest S., Rivier C. (1994). Stress and interleukin-1 beta-induced activation of c-fos, NGFI-B and CRF gene expression in the hypothalamic PVN: Comparison between Sprague-Dawley, Fisher-344 and Lewis rats.. J. Neuroendocrinol..

[59] Karalis K., Crofford L., Wilder R.L., Chrousos G.P. (1995). Glucocorticoid and/or glucocorticoid antagonist effects in inflammatory disease- susceptible Lewis rats and inflammatory disease-resistant Fischer rats.. Endocrinology.

[60] Marissal-Arvy N., Gaumont A., Langlois A., Dabertrand F., Bouchecareilh M., Tridon C., Mormede P. (2007). Strain differences in hypothalamic-pituitary-adrenocortical axis function and adipogenic effects of corticosterone in rats.. J. Endocrinol..

[61] Gómez F., De Kloet E.R., Armario A. (1998). Glucocorticoid negative feedback on the HPA axis in five inbred rat strains.. Am. J. Physiol. Regul. Integr. Comp. Physiol..

[62] Stefferl A., Linington C., Holsboer F., Reul J.M.H.M. (1999). Susceptibility and resistance to experimental allergic encephalomyelitis: relationship with hypothalamic-pituitary-adrenocortical axis responsiveness in the rat.. Endocrinology.

[63] Oitzl M.S., van Haarst A.D., Sutanto W., Ron de Kloet E. (1995). Corticosterone, brain mineralocorticoid receptors (MRS) and the activity of the hypothalamic-pituitary-adrenal (hpa) axis: The Lewis rat as an example of increased central MR capacity and a hyporesponsive HPA axis.. Psychoneuroendocrinology.

[64] Pardon M.C., Gould G.G., Garcia A., Phillips L., Cook M.C., Miller S.A., Mason P.A., Morilak D.A. (2002). Stress reactivity of the brain noradrenergic system in three rat strains differing in their neuroendocrine and behavioral responses to stress: Implications for susceptibility to stress-related neuropsychiatric disorders.. Neuroscience.

[65] Klenerova V., Sida P., Hynie S., Jurcovicova J. (2001). Rat strain differences in responses of plasma prolactin and PRL mRNA expression after acute amphetamine treatment or restraint stress.. Cell. Mol. Neurobiol..

[66] (2006). Trnečková, L.; Armario, A.; Hynie, S.; Šída, P.; Klenerová, V. Differences in the brain expression of c-fos mRNA after restraint stress in Lewis compared to Sprague–Dawley rats.. Brain Res..

[67] Sarrieau A., Mormède P. (1998). Hypothalamic-pituitary-adrenal axis activity in the inbred brown Norway and Fischer 344 rat strains.. Life Sci..

[68] Uchida S., Nishida A., Hara K., Kamemoto T., Suetsugi M., Fujimoto M., Watanuki T., Wakabayashi Y., Otsuki K., McEwen B.S., Watanabe Y. (2008). Characterization of the vulnerability to repeated stress in Fischer 344 rats: Possible involvement of microRNA- mediated down-regulation of the glucocorticoid receptor.. Eur. J. Neurosci..

[69] Broadhurst P.L. (1975). The Maudsley Reactive and Nonreactive strains of rats: A survey.. Behav. Genet..

[70] Blizard D.A. (1981). The Maudsley reactive and nonreactive strains: A North American perspective.. Behav. Genet..

[71] Overstreet D.H., Rezvani A.H., Janowsky D.S. (1992). Maudsley reactive and nonreactive rats differ only in some tasks reflecting emotionality.. Physiol. Behav..

[72] Paterson A., Whiting P.J., Gray J.A., Flint J., Dawson G.R. (2001). Lack of consistent behavioural effects of Maudsley reactive and non-reactive rats in a number of animal tests of anxiety and activity.. Psychopharmacology.

[73] Abel E.L. (1991). Behavior and corticosteroid response of maudsley reactive and nonreactive rats in the open field and forced swimming test.. Physiol. Behav..

[74] Buda M., Lachuer J., Devauges V., Barbagli B., Blizard D., Sara S.J. (1994). Central noradrenergic reactivity to stress in Maudsley rat strains.. Neurosci. Lett..

[75] Blizard D.A., Eldridge J.C., Jones B.C. (2015). The defecation index as a measure of emotionality: Questions raised by HPA axis and prolactin response to stress in the maudsley model.. Behav. Genet..

[76] Kosti O., Raven P.W., Renshaw D., Hinson J.P. (2006). Intra-adrenal mechanisms in the response to chronic stress: Investigation in a rat model of emotionality.. J. Endocrinol..

[77] Liebsch G., Montkowski A., Holsboer F., Landgraf R. (1998). Behavioural profiles of two Wistar rat lines selectively bred for high or low anxiety-related behaviour.. Behav. Brain Res..

[78] Landgraf R., Wigger A., Holsboer F., Neumann I.D. (1999). Hyperreactive hypothalamo-pituitary-adrenocortical axis in rats bred for high anxiety-related behaviour.. J. Neuroendocrinol..

[79] Keck M.E., Welt T., Müller M.B., Uhr M., Ohl F., Wigger A., Toschi N., Holsboer F., Landgraf R. (2003). Reduction of hypothalamic vasopressinergic hyperdrive contributes to clinically relevant behavioral and neuroendocrine effects of chronic paroxetine treatment in a psychopathological rat model.. Neuropsychopharmacology.

[80] Frank E., Salchner P., Aldag J.M., Salomé N., Singewald N., Landgraf R., Wigger A. (2006). Genetic predisposition to anxiety-related behavior determines coping style, neuroendocrine responses, and neuronal activation during social defeat.. Behav. Neurosci..

[81] Brush F.R., Baron S., Froehlich J.C., Ison J.R., Pellegrino L.J., Phillips D.S., Sakellaris P.C., Williams V.N. (1985). Genetic differences in avoidance learning by Rattus norvegicus: Escape/avoidance responding, sensitivity to electric shock, discrimination learning, and open-field behavior.. J. Comp. Psychol..

[82] Brush F.R., del Paine S.N., Pellegrino L.J., Rykaszewski I.M., Dess N.K., Collins P.Y. (1988). CER suppression, passive-avoidance learning, and stress-induced suppression of drinking in the Syracuse high- and low-avoidance strains of rats (Rattus norvegicus).. J. Comp. Psychol..

[83] Brush F.R. (2003). Selection for differences in avoidance learning: The Syracuse strains differ in anxiety, not learning ability.. Behav. Genet..

[84] Del Paine S.N., Brush F.R. (1990). Adrenal morphometry in unilateral and sham adrenalectomized syracuse high and low avoidance rats.. Physiol. Behav..

[85] Brush F.R., Isaacson M.D., Pellegrino L.J., Rykaszewski I.M., Shain C.N. (1991). Characteristics of the pituitary-adrenal system in the syracuse high-and low-avoidance strains of rats (Rattus norvegicus).. Behav. Genet..

[86] Gupta P., Brush F.R. (1998). Differential behavioral and endocrinological effects of corticotropin-releasing hormone (CRH) in the Syracuse high- and low-avoidance rats.. Horm. Behav..

[87] Ohta R., Matsumoto A., Hashimoto Y., Nagao T., Mizutani M. (1995). Behavioral characteristics of rats selectively bred for high and low avoidance shuttlebox response.. Congenit. Anom..

[88] Ohta R., Matsumoto A., Nagao T., Mizutani M. (1998). Comparative study of behavioral development between high and low shuttlebox avoidance rats.. Physiol. Behav..

[89] Ohta R., Shirota M., Adachi T., Tohei A., Taya K. (1999). Plasma ACTH levels during early, two-way avoidance acquisition in highand low-avoidance rats (Hatano strains).. Behav. Genet..

[90] Asai S., Ohta R., Shirota M., Watanabe G., Taya K. (2004). Differential responses of the hypothalamo-pituitary-adrenocortical axis to acute restraint stress in Hatano high- and low-avoidance rats.. J. Endocrinol..

[91] Jaroenporn S., Nagaoka K., Ohta R., Shirota M., Watanabe G., Taya K. (2009). Differences in adrenocortical secretory and gene expression responses to stimulation in vitro by ACTH or prolactin between high- and low-avoidance Hatano rats.. Stress.

[92] Akieda-Asai S., Ohta R., Shirota M., Jaroenporn S., Watanabe G., Taya K. (2011). Endocrinological differences between Hatano highand low-avoidance rats during early two-way avoidance acquisition.. Exp. Anim..

[93] Bignami G. (1965). Selection for high rates and low rates of avoidance conditioning in the rat.. Anim. Behav..

[94] Steimer T., Driscoll P. (2003). Divergent stress responses and coping styles in psychogenetically selected Roman high-(RHA) and low- (RLA) avoidance rats: Behavioural, neuroendocrine and developmental aspects.. Stress.

[95] Walker C.D., Rivest R.W., Meaney M.J., Aubert M.L. (1989). Differential activation of the pituitary-adrenocortical axis after stress in the rat: Use of two genetically selected lines (Roman low- and highavoidance rats) as a model.. J. Endocrinol..

[96] Gentsch C., Lichtsteiner M., Feer H. (1981). Locomotor activity, defecation score and corticosterone levels during an openfield exposure: A comparison among individually and group-housed rats, and genetically selected rat lines.. Physiol. Behav..

[97] Castanon N., Dulluc J., Le Moal M., Mormède P. (1992). Prolactin as a link between behavioral and immune differences between the Roman rat lines.. Physiol. Behav..

[98] Steimer T., Escorihuela R.M., Fernández-teruel A., Driscoll P. (1998). Long‐term behavioural and neuroendocrine changes in roman HIGH‐(RHA/Verh) and LOW‐(RLA‐Verh) avoidance rats following neonatal handling.. Int. J. Dev. Neurosci..

[99] Aubry J.M., Bartanusz V., Driscoll P., Schulz P., Steimer T., Kiss J.Z. (1995). Corticotropin-releasing factor and vasopressin mRNA levels in roman high and low-avoidance rats: Response to openfield exposure.. Neuroendocrinology.

[100] Carrasco J., Márquez C., Nadal R., Tobeña A., Fernández-Teruel A., Armario A. (2008). Characterization of central and peripheral components of the hypothalamus–pituitary–adrenal axis in the inbred Roman rat strains.. Psychoneuroendocrinology.

[101] Díaz-Morán S., Palència M., Mont-Cardona C., Cañete T., Blázquez G., Martínez-Membrives E., López-Aumatell R., Tobeña A., Fernández-Teruel A. (2012). Coping style and stress hormone responses in genetically heterogeneous rats: Comparison with the Roman rat strains.. Behav. Brain Res..

[102] Okamoto K., Aoki K. (1963). Development of a strain of spontaneously hypertensive rats.. Jpn. Circ. J..

[103] Werner S.C., Manger W.M., Radichevich I., Wolff M., Estorff I.V. (1975). Excessive thyrotropin concentrations in the circulation of the spontaneously hypertensive rat.. Exp. Biol. Med..

[104] Regan S.L., Williams M.T., Vorhees C.V. (2022). Review of rodent models of attention deficit hyperactivity disorder.. Neurosci. Biobehav. Rev..

[105] Aleksandrova L.R., Wang Y.T., Phillips A.G. (2019). Evaluation of the Wistar-Kyoto rat model of depression and the role of synaptic plasticity in depression and antidepressant response.. Neurosci. Biobehav. Rev..

[106] Paré W.P., Redei E. (1993). Depressive behavior and stress ulcer in Wistar Kyoto rats.. J. Physiol. Paris.

[107] Lahmame A., Gomez F., Armario A. (1996). Fawn-hooded rats show enhanced active behaviour in the forced swimming test, with no evidence for pituitary-adrenal axis hyperactivity.. Psychopharmacology (Berl.).

[108] Lahmame A., del Arco C., Pazos A., Yritia M., Armario A. (1997). Are Wistar-Kyoto rats a genetic animal model of depression resistant to antidepressants?. Eur. J. Pharmacol..

[109] McCarty R. (1983). Stress, behavior and experimental hypertension.. Neurosci. Biobehav. Rev..

[110] Hendley E.D., Cierpial M.A., McCarty R. (1988). Sympathetic-adrenal medullary response to stress in hyperactive and hypertensive rats.. Physiol. Behav..

[111] Zhang T., Reid K., Acuff C.G., Jin C.B., Rockhold R.W. (1994). Cardiovascular and analgesic effects of a highly palatable diet in spontaneously hypertensive and Wistar-Kyoto rats.. Pharmacol. Biochem. Behav..

[112] Durand M., Berton O., Aguerre S., Edno L., Combourieu I., Mormède P., Chaouloff F. (1999). Effects of repeated fluoxetine on anxiety- related behaviours, central serotonergic systems, and the corticotropic axis in SHR and WKY rats.. Neuropharmacology.

[113] Lim D.Y., Jang S.J., Park D.G. (2002). Comparison of catecholamine release in the isolated adrenal glands of SHR and WKY rats.. Auton. Autacoid Pharmacol..

[114] McBride S.M., Culver B., Flynn F.W. (2006). Prenatal and early postnatal dietary sodium restriction sensitizes the adult rat to amphetamines.. Am. J. Physiol. Regul. Integr. Comp. Physiol..

[115] (2019). Vavřínová, A.; Behuliak, M.; Bencze, M.; Vodička, M.; Ergang, P.;
Vaněčková, I.; Zicha, J. Sympathectomy-induced blood pressure reduction in adult normotensive and hypertensive rats is counteracted by enhanced cardiovascular sensitivity to vasoconstrictors.. Hypertens. Res..

[116] Nickerson P.A. (1976). The adrenal cortex in spontaneously hypertensive rats. A quantitative ultrastructural study.. Am. J. Pathol..

[117] Nishiyama K., Nishiyama A., Frohlich E.D. (1976). Regional blood flow in normotensive and spontaneously hypertensive rats.. Am. J. Physiol..

[118] Ayachi S. (1979). Increased dietary calcium lowers blood pressure in the spontaneously hypertensive rat.. Metabolism.

[119] Häusler A., Girard J., Baumann J.B., Ruch W., Otten U.H. (1983). Long-term effects of betamethasone on blood pressure and hypothalamo- pituitary-adrenocortical function in spontaneously hypertensive and normotensive rats.. Horm. Res..

[120] Paré W.P., Schimmel G.T. (1986). Stress ulcer in normotensive and spontaneously hypertensive rats.. Physiol. Behav..

[121] Fukuda S., Tsuchikura S., Iida H. (2004). Age-related changes in blood pressure, hematological values, concentrations of serum biochemical constituents and weights of organs in the SHR/Izm, SHRSP/Izm and WKY/Izm.. Exp. Anim..

[122] Gilad G.M., Jimerson D.C. (1981). Modes of adaptation of peripheral neuroendocrine mechanisms of the sympatho-adrenal system to short-term stress as studied in two inbred rat strains.. Brain Res..

[123] Harrap S.B., Louis W.J., Doyle A.E. (1984). Failure of psychosocial stress to induce chronic hypertension in the rat.. J. Hypertens..

[124] McCarty R., Kvetnansky R., Raymond Lake C., Thoa N.B., Kopin I.J. (1978). Sympatho-adrenal activity of SHR and WKY rats during recovery from forced immobilization.. Physiol. Behav..

[125] Sowers J., Tuck M., Asp N.D., Sollars E. (1981). Plasma aldosterone and corticosterone responses to adrenocorticotropin, angiotensin, potassium, and stress in spontaneously hypertensive rats.. Endocrinology.

[126] Hashimoto K., Makino S., Hirasawa R., Takao T., Sugawara M., Murakami K., Ono K., Ota Z. (1989). Abnormalities in the hypothalamo- pituitary-adrenal axis in spontaneously hypertensive rats during development of hypertension.. Endocrinology.

[127] Freeman R.H., Davis J.O., Aharon N.V., Ulick S., Weinberger M.H. (1975). Control of aldosterone secretion in the spontaneously hypertensive rat.. Circ. Res..

[128] DeVito W.J., Sutterer J.R., Robert Brush F. (1981). The pituitary-adrenal response to ether stress in the spontaneously hypertensive and normotensive rat.. Life Sci..

[129] Komanicky P., Reiss D.L., Dale S.L., Melby J.C. (1982). Role of adrenal steroidogenesis in etiology of hypertension in the spontaneously hypertensive rat.. Endocrinology.

[130] Krukoff T.L., MacTavish D., Jhamandas J.H. (1999). Hypertensive rats exhibit heightened expression of corticotropin-releasing factor in activated central neurons in response to restraint stress.. Brain Res. Mol. Brain Res..

[131] Autelitano D.J., Van Den Buuse M. (1997). Concomitant up-regulation of proopiomelanocortin and dopamine D2-receptor gene expression in the pituitary intermediate lobe of the spontaneously hypertensive rat.. J. Neuroendocrinol..

[132] Braas K.M., Hendley E.D., May V., Cronin K.M., McAuley J.A. (1994). Anterior pituitary proopiomelanocortin expression is decreased in hypertensive rat strains.. Endocrinology.

[133] Häusler A., Girard J., Baumann J.B., Ruch W., Otten U.H. (1983). Stress-induced secretion of ACTH and corticosterone during development of spontaneous hypertension in rats.. Clin. Exp. Hypertens. A.

[134] Kvetnansky R., McCarty R., Thoa N.B., Lake C.R., Kopin I.J. (1979). Sympatho-adrenal responses of spontaneously hypertensive rats to immobilization stress.. Am. J. Physiol. Heart Circ. Physiol..

[135] Chiueh C.C., McCarty R. (1981). Sympatho-adrenal hyperreactivity to footshock stress but not to cold exposure in spontaneously hypertensive rats.. Physiol. Behav..

[136] Knardahl S., Murison R. (1989). Plasma corticosterone and renin activity during two-way active avoidance learning in spontaneously hypertensive and Wistar-Kyoto rats.. Behav. Neural Biol..

[137] Solberg L.C., Olson S.L., Turek F.W., Redei E. (2001). Altered hormone levels and circadian rhythm of activity in the WKY rat, a putative animal model of depression.. Am. J. Physiol. Regul. Integr. Comp. Physiol..

[138] Hauger R.L., Shelat S.G., Redei E.E. (2002). Decreased corticotropinreleasing factor receptor expression and adrenocorticotropic hormone responsiveness in anterior pituitary cells of Wistar-Kyoto rats.. J. Neuroendocrinol..

[139] Shepard J., Myers D. (2008). Strain differences in anxiety-like behavior: Association with corticotropin-releasing factor.. Behav. Brain Res..

[140] Bravo J.A., Dinan T.G., Cryan J.F. (2011). Alterations in the central CRF system of two different rat models of comorbid depression and functional gastrointestinal disorders.. Int. J. Neuropsychopharmacol..

[141] Rittenhouse P.A., López-Rubalcava C., Stanwood G.D., Lucki I. (2002). Amplified behavioral and endocrine responses to forced swim stress in the Wistar-Kyoto rat.. Psychoneuroendocrinology.

[142] Redei E., Paré W.P., Aird F., Kluczynski J. (1994). Strain differences in hypothalamic-pituitary-adrenal activity and stress ulcer.. Am. J. Physiol. Regul. Integr. Comp. Physiol..

[143] Braley L.M., Menachery A., Williams G.H. (1983). Specificity of the alteration in aldosterone biosynthesis in the spontaneously hypertensive rat.. Endocrinology.

[144] Slezak P., Puzserova A., Balis P., Sestakova N., Majzunova M., Dovinova I., Kluknavsky M., Bernatova I. (2014). Genotype-related effect of crowding stress on blood pressure and vascular function in young female rats.. BioMed Res. Int..

[145] Overstreet D.H., Wegener G. (2013). The flinders sensitive line rat model of depression - 25 years and still producing.. Pharmacol. Rev..

[146] Overstreet D.H., Booth R.A., Dana R., Risch S.C., Janowsky D.S. (1986). Enhanced elevation of corticosterone following arecoline administration to rats selectively bred for increased cholinergic function.. Psychopharmacology.

[147] Owens M.J., Overstreet D.H., Knight D.L., Rezvani A.H., Ritchie J.C., Bissette G., Janowsky D.S., Nemeroff C.B. (1991). Alterations in the hypothalamic-pituitary-adrenal axis in a proposed animal model of depression with genetic muscarinic supersensitivity.. Neuropsychopharmacology.

[148] Ayensu W.K., Pucilowski O., Mason G.A., Overstreet D.H., Rezvani A.H., Janowsky D.S. (1995). Effects of chronic mild stress on serum complement activity, saccharin preference, and corticosterone levels in Flinders lines of rats.. Physiol. Behav..

[149] Elsenbruch S., Wang L., Hollerbach S., Schedlowski M., Tougas G. (2004). Pseudo-affective visceromotor responses and HPA axis activation following colorectal distension in rats with increased cholinergic sensitivity.. Neurogastroenterol. Motil..

[150] Mattsson H., Arani Z., Astin M., Bayati A., Overstreet D.H., Lehmann A. (2005). Altered neuroendocrine response and gastric dysmotility in the flinders sensitive line rat.. Neurogastroenterol. Motil..

[151] Malkesman O., Braw Y., Maayan R., Weizman A., Overstreet D.H., Shabat-Simon M., Kesner Y., Touati-Werner D., Yadid G., Weller A. (2006). Two different putative genetic animal models of childhood depression.. Biol. Psychiatry.

[152] Malkesman O., Maayan R., Weizman A., Weller A. (2006). Aggressive behavior and HPA axis hormones after social isolation in adult rats of two different genetic animal models for depression.. Behav. Brain Res..

[153] Braw Y., Malkesman O., Merlender A., Bercovich A., Dagan M., Maayan R., Weizman A., Weller A. (2006). Stress hormones and emotion-regulation in two genetic animal models of depression.. Psychoneuroendocrinology.

[154] Thiele S., Spehl T.S., Frings L., Braun F., Ferch M., Rezvani A.H., Furlanetti L.L., Meyer P.T., Coenen V.A., Döbrössy M.D. (2016). Long-term characterization of the Flinders Sensitive Line rodent model of human depression: Behavioral and PET evidence of a dysfunctional entorhinal cortex.. Behav. Brain Res..

[155] Mncube K., Möller M., Harvey B.H. (2021). Post-weaning social isolated flinders sensitive line rats display bio-behavioural manifestations resistant to fluoxetine: A model of treatment-resistant depression.. Front. Psychiatry.

[156] Kokras N., Sotiropoulos I., Pitychoutis P.M., Almeida O.F.X., Papadopoulou-Daifoti Z. (2011). Citalopram-mediated anxiolysis and differing neurobiological responses in both sexes of a genetic model of depression.. Neuroscience.

[157] Walker S.E., Zanoletti O., Guillot de Suduiraut I., Sandi C. (2017). Constitutive differences in glucocorticoid responsiveness to stress are related to variation in aggression and anxiety-related behaviors.. Psychoneuroendocrinology.

[158] Huzard D., Ghosal S., Grosse J., Carnevali L., Sgoifo A., Sandi C. (2019). Low vagal tone in two rat models of psychopathology involving high or low corticosterone stress responses.. Psychoneuroendocrinology.

[159] Walker S.E., Sandi C. (2018). Long-term programing of psychopathology-like behaviors in male rats by peripubertal stress depends on individual’s glucocorticoid responsiveness to stress.. Stress.

[160] Huzard D., Vouros A., Monari S., Astori S., Vasilaki E., Sandi C. (2020). Constitutive differences in glucocorticoid responsiveness are related to divergent spatial information processing abilities.. Stress.

[161] Elenkov I.J., Kvetnansky R., Hashiramoto A., Bakalov V.K., Link A.A., Zachman K., Crane M., Jezova D., Rovensky J., Dimitrov M.A., Gold P.W., Bonini S., Fleisher T., Chrousos G.P., Wilder R.L. (2008). Low versus high-baseline epinephrine output shapes opposite innate cytokine profiles: Presence of Lewis and Fischer-like neurohormonal immune phenotypes in humans?. J. Immunol..

[162] Goldstein D.S., Garty M., Bagdy G., Szemeredi K., Sternberg E.M., Listwak S., Pacak K., Deka-Starosta A., Hoffman A., Chang P.C., Stull R., Gold P.W., Kopin I.J. (1993). Role of CRH in glucopenia-induced adrenomedullary activation in rats.. J. Neuroendocrinol..

[163] Boersma G.J., Scheurink A.J.W., Wielinga P.Y., Steimer T.J., Benthem L. (2009). The passive coping Roman Low Avoidance rat, a nonobese rat model for insulin resistance.. Physiol. Behav..

[164] Castanon N., Dulluc J., Le Moal M., Mormède P. (1994). Maturation of the behavioral and neuroendocrine differences between the Roman rat lines.. Physiol. Behav..

[165] Steimer T., la Fleur S., Schulz P.E. (1997). Neuroendocrine correlates of emotional reactivity and coping in male rats from the Roman high (RHA/Verh)- and low (RLA/Verh)-avoidance lines.. Behav. Genet..

[166] Steger R.W., Avila-Jimenez R., Amador A., Johns A. (1984). Altered hypothalamic monoamine metabolism and pituitary prolactin regulation in female spontaneously hypertensive rats.. Life Sci..

[167] Amador A., Steger R.W., Bartke A., Johns A., Hayashi R.H., Stallings M.H. (1983). Pituitary and testicular function in spontaneously hypertensive rats.. J. Androl..

[168] Sanchís-Ollé M., Sánchez-Benito L., Fuentes S., Gagliano H., Belda X., Molina P., Carrasco J., Nadal R., Armario A. (2021). Male long-Evans rats: An outbred model of marked hypothalamicpituitary-adrenal hyperactivity.. Neurobiol. Stress.

[169] Jurcovicová J., Vigas M., Klír P., Jezová D. (1984). Response of prolactin, growth hormone and corticosterone secretion to morphine administration or stress exposure in Wistar-AVN and Long Evans rats.. Endocrinol. Exp..

[170] (2020). Vodička, M.; Vavřínová, A.; Mikulecká, A.; Zicha, J.; Behuliak,
M. Hyper-reactivity of HPA axis in Fischer 344 rats is associated with impaired cardiovascular and behavioral adaptation to repeated restraint stress.. Stress.

[171] Márquez C., Nadal R., Armario A. (2005). Responsiveness of the hypothalamic-pituitary-adrenal axis to different novel environments is a consistent individual trait in adult male outbred rats.. Psychoneuroendocrinology.

[172] Márquez C., Nadal R., Armario A. (2006). Influence of reactivity to novelty and anxiety on hypothalamic-pituitary-adrenal and prolactin responses to two different novel environments in adult male rats.. Behav. Brain Res..

[173] Nadal R., Gabriel-Salazar M., Sanchís-Ollé M., Gagliano H., Belda X., Armario A. (2021). Individual differences in the neuroendocrine response of male rats to emotional stressors are not trait-like and strongly depend on the intensity of the stressors.. Psychoneuroendocrinology.

[174] Armario A. (2021). The forced swim test: Historical, conceptual and methodological considerations and its relationship with individual behavioral traits.. Neurosci. Biobehav. Rev..

[175] Will C.C., Aird F., Redei E.E. (2003). Selectively bred Wistar–Kyoto rats: An animal model of depression and hyper-responsiveness to antidepressants.. Mol. Psychiatry.

[176] Solberg L.C., Ahmadiyeh N., Baum A.E., Vitaterna M.H., Takahashi J.S., Turek F.W., Redei E.E. (2003). Depressive-like behavior and stress reactivity are independent traits in a Wistar Kyoto × Fisher 344 cross.. Mol. Psychiatry.

